# Investigation of pilots' mental health and analysis of influencing factors in China: based on structural equation model

**DOI:** 10.1186/s12889-022-13764-y

**Published:** 2022-07-15

**Authors:** Feifei Yu, Xuxia Li, Jishun Yang

**Affiliations:** PLA Naval Medical Center, Naval Medical University (Second Military Medical University), Yangpu District, 800 Xiangyin Road, Shanghai, 200433 China

**Keywords:** Influencing factors, Health-related quality of life, Physical health, Mental health, Pilot

## Abstract

**Background:**

Pilots' physical and mental health might be significant contributing factors to flight safety. Exploring pilots’ health-related quality of life (HRQoL) is crucial for aviation security, health management, and psychological security. This study aimed to explore HRQoL and mental health of pilots and analyze the health characteristics and influencing factors, such as demographic data, personality traits, social support, and resilience. It may provide data for a theoretical basis for aviation security work and health management strategy.

**Methods:**

This is a cross-sectional study using quantitative approaches. Two hundred twenty male pilots with an average age of 33.31 years participated. They answered a social demographic questionnaire, Symptom Checklist-90**,** Short Form 36 Health Survey Questionnaire, Perceived social support scale, Connor-Davidson resilience scale, and Big Five Personality Inventories, whose data were analyzed using descriptive and inferential statistics.

**Results:**

The mediating effect of personality factors between resilience and the HRQoL of pilots was observed. Personality factors also mediated the relationship between social support and the mental health of pilots.

**Conclusion:**

Pilots’ mental health and quality of life need to be taken seriously. Social support, resilience, and personality factors affect pilots’ mental health and quality of life.

## Introduction

Pilots' physical and mental health are significant factors for flight safety. The medical fitness of pilots is part of the civil aviation safety scenery, and psychological state is essential for flight safety [1, 2]. Stricter requirements for pilots’ physical and psychological functions of pilots are necessary. For example, a survey of professional pilots’ health and well-being analyzed by Marion Venus found that significant psychosocial stress was associated with pilots’ jobs and livelihood [3]. Meanwhile, an investigation done in Germany detected acute effects on fatigue, workload, recovery, and performance after consecutive short-haul operations [4]. Therefore, exploring factors affecting pilots’ physical and mental health has become critical for aviation security, health management, and psychological security.

*Resilience* is the ability to save, recover and even improve oneself after facing adversity and some overwhelming disasters and may be closely associated with mental health [5]. It allows one to bounce back from adverse life events and function normally, using self-regulation and cognitive coping skills when faced with stressful situations to reduce the deleterious effects on the individual and maintain their well-being. However, interpersonal and contextual factors, for example, the characteristics of the environment, could moderate the link between individual characteristics and mental wellness [5]. A scale emphasizing individual self-understanding and self-feeling about social support could measure these interpersonal and contextual factors. It assesses the individual’s perceived level of social support from various sources, such as family, friends, and others. The total score reflects the individual’s sense of social support from all sources.

WHO defines *health* as “a state of complete physical, mental and social well-being, and not merely the absence of disease or infirmity” (https://www.who.int/director-general/speeches/detail/assembly-of-parties-of-the-international-development-law-organization). According to the Center for Disease Control and Prevention (CDC) definition, *health-related quality of life* (HRQoL) is an individual’s or group’s perceived physical and mental health over time (https://www.cdc.gov/hrqol/). This study investigates the HRQoL of pilots, primarily related to physical and mental health, and analyzes characteristics of and influencing factors on pilots from the perspectives of demographic data, personality traits, social support, and resilience. The study aims to provide a theoretical basis for aviation security work and health management strategy.

## Methods

### Participants

From July to September 2017, 250 questionnaires were distributed to pilots in different regions of China. Two hundred twenty were recovered, resulting in a final effective rate of 88.0%. The average age of the sample was 33.31 ± 7.27 years. All participants were male due to the very low proportion of female pilots in China. Table 1 shows the basic information about pilots.

**Table 1:** Demographic characteristics of participants (*N* = 220)

Subject	Group	Frequency	Percentage
Years of working	< 5 years	21	9.5
	5 ~ 10 years	99	45.0
	> 10 years	100	45.5
Marital status	Unmarried	57	25.9
	Married	154	70.0
	Divorced	9	4.1
Only child	Yes	104	47.7
	No	116	52.3
Education degree	Junior college	23	10.5
	Undergraduate	189	85.9
	Master degree or above	8	3.6
Census register	Urban residence	105	47.7
	Rural residence	115	52.3

### Materials

#### The Perceived Social Support Scale (PSSS)

The PSSS was developed by Zimet to evaluate the understanding and utilization of support derived from family, friends, and other important social relationships [6]. Blumental subsequently revised it. The Chinese version was translated and revised by Jiang. It provides high reliability and validity in this study. The scale contains 12 items, using Likert 7-level scoring, from 1 point (*strongly disagree*) to 7 points (*strongly agree*). The scale includes three subscales including *family*, *social* and *other support*. Higher scores indicate robust social support systems. Scores below 32 indicate low social support levels. Scores over 50 indicate good social support systems [7].

#### The Connor-Davidson resilience scale (CD-RISC)

The Chinese version of the scale was revised by Yu to assess resilience, specifically, the ability to cope with adversity. The 25-item scale contains three conceptually distinct subscales, including *strength*, *tenacity*, and *optimism*. Responses are measured on a 5-point Likert scale ranging from 0 (*not true at all*) to 4 (*true nearly all the time*), with higher total scores denoting strong resilience. This scale has high reliability and validity [8, 9].

#### The Big Five Personality Inventory (BFI-44)

The Chinese version of the BFI-44 was revised by John and Srivastava. It measures individuals’ central personality traits. The 44-item scale contains five subscales: *extraversion*, *agreeableness*, *conscientiousness*, *neuroticism*, and *openness to experience*. Likert 5-point scale scoring is used, from 1 (*strongly disagree*) to 5 points (*strongly agree*). This scale shows high reliability and validity [10].

#### The symptom checklist-90 (SCL-90)

The SCL-90 was developed and revised by Derogatis. It uses nine dimensions to measure individual mental health. The scale contains 90 items. They assess *somatization*, *obsessive symptoms*, *interpersonal sensitivity*, *depression*, *anxiety*, *hostility*, *terror*, *paranoia*, and *psychosis*. This scale was scored on a 5-point scale, from 0 (*no such symptom*) to 4 points (*serious*). The Chinese version of the scale is widely used and has high reliability and validity [11].

#### The Short Form 36 health Survey Questionnaire (SF-36)

The SF-36 was compiled by the Boston Health Institute to measure individual health-related quality of life (HRQoL). The questionnaire comprises 36 items, including nine multiple-item subscales that evaluate the *physical function*, *physical role*, *bodily pain*, *general health*, *vitality*, *social functioning*, *role-emotion*, *mental health*, and *reported health transition*. The questionnaire demonstrates high reliability and validity. The first four dimensions were used to evaluate the physical health of pilots [12].

### Statistical analyses

SPSS Version 23 was used for descriptive statistics, correlation analysis, and regression analysis. AMOS Version 17.0 was used to establish and optimize the structural equation. One-way variance analysis (ANOVA) was performed to compare the physical and psychological health of pilots related to demographic factors. Pearson correlation analysis was used to measure relationships between variables. Then, a multiple hierarchical regression analysis was performed. Finally, using structural equations, the influence paths and factors’ effect sizes were examined.

## Results

### Differences in pilots' HRQoL related to demographic variables

Using the demographic variables number of years of employment, marital status, only child status, educational level, and census register as factors, the HRQoL of pilots were compared. Table 2 shows the results of these comparisons. Significant differences were detected in physical function related to educational level (*F* = 13.853, *p* < 0.001). Next, a post-hoc test was conducted. The pairwise comparison results showed that the physical functioning of pilots with undergraduate, master’s degrees or above was better than that of pilots with only junior college education (*LSD-t* = 17.675, *p* < 0.001; *LSD-t* = 21.630, *p* = 0.001). However, no significant differences were found between the undergraduate and master's degrees or above (*LSD-t* = 3.955, *p* = 0.481). In addition, the general health of urban pilots was better than that of rural ones (*F* = 5.426, *p* = 0.021). Differences between other pilots’ HRQoL indicators related to demographic variables were not significant.

**Table 2:** Physical health of pilots in demographic variables (*N* = 220)

Subject	Group	Physical function	Physical role	Bodily pain	General health
Years of working	< 5 years	97.62 ± 5.61	92.86 ± 19.59	69.52 ± 13.59	83.81 ± 19.48
	5 ~ 10 years	92.42 ± 15.55	87.88 ± 27.74	69.39 ± 13.98	74.65 ± 20.62
	> 10 years	90.05 ± 18.40	84.50 ± 31.13	68.90 ± 13.02	76.60 ± 18.66
	*F*	1.979	0.857	0.041	1.893
	*p*	0.141	0.426	0.960	0.153
Marital status	Unmarried	94.74 ± 14.65	90.35 ± 25.77	69.47 ± 13.81	80.09 ± 18.69
	Married	90.42 ± 17.29	85.06 ± 30.32	68.96 ± 12.99	75.39 ± 19.97
	Divorced	97.78 ± 2.635	94.44 ± 11.02	71.11 ± 19.65	70.56 ± 20.53
	*F*	2.074	1.036	0.126	1.602
	*p*	0.128	0.356	0.882	0.204
Only child	Yes	93.65 ± 13.51	89.18 ± 25.78	70.19 ± 11.74	77.16 ± 19.98
	No	90.22 ± 18.53	84.70 ± 31.06	68.28 ± 14.82	75.73 ± 19.55
	*F*	2.425	1.340	1.113	0.288
	*p*	0.121	0.248	0.293	0.592
Education degree	Junior college	75.87 ± 28.39	77.17 ± 36.86	66.96 ± 11.84	68.91 ± 14.37
	Undergraduate	93.54 ± 13.54	88.10 ± 27.72	69.42 ± 13.26	77.59 ± 19.85
	Master degree or above	97.50 ± 2.67	84.38 ± 22.90	70.00 ± 22.04	70.00 ± 25.91
	*F*	13.853	1.521	0.356	2.455
	*p*	< 0.001	0.221	0.701	0.088
Census register	Urban residence	92.76 ± 14.14	89.52 ± 25.66	70.00 ± 11.68	79.62 ± 17.79
	Rural residence	91.00 ± 18.25	84.35 ± 31.14	68.43 ± 14.90	73.48 ± 20.98
	*F*	0.632	1.790	0.742	5.427
	*p*	0.427	0.182	0.390	0.021*

^***^p < 0.001, **p < 0.01, *p < 0.05

With years of employment, marital and only child status, educational level, and census register as factors, the mental health of pilots related to demographic variables was compared. Table 3 shows there were significant differences in somatization related to educational level (*F* = 3.133, *p* = 0.046). Then post-hoc testing was conducted. The pairwise comparison results showed that the somatization of pilots with fewer than five years of work experience was less severe than that of pilots employed for between 5 and10 years and more than 10 years (*LSD-t* = 0.116, *p* = 0.047; *LSD-t* = 0.145, *p* = 0.013). However, there was no significant difference between 5–10 years and more than 10 years (*LSD-t* = 0.029, *p* = 0.396). In addition, the somatization, anxiety, and terror levels of only-child pilots were lower than those of non-only-child pilots (*F* = 4.900, *p* = 0.028; *F* = 4.754, *p* = 0.030; *F* = 4.460, *p* = 0.036). The anxiety level of urban pilots was better than that of rural ones (*F* = 4.795, *p* = 0.030). Differences in other pilots’ mental health indicators related to demographic variables were not significant.

**Table 3:** Mental health of pilots in demographic variables (*N* = 220)

Subject	Group	Somatization	Obsessive symptoms	Interpersonal sensitivity	Depression	Anxiety	Hostility	Terror	Paranoia	Psychosis
Years of working	< 5 years	0.03 ± 0.08	0.13 ± 0.35	0.11 ± 0.32	0.10 ± 0.35	0.06 ± 0.21	0.04 ± 0.12	0.03 ± 0.10	0.07 ± 0.22	0.07 ± 0.24
	5 ~ 10 years	0.15 ± 0.26	0.17 ± 0.24	0.15 ± 0.26	0.13 ± 0.26	0.11 ± 0.20	0.13 ± 0.26	0.07 ± 0.13	0.15 ± 0.28	0.13 ± 0.23
	> 10 years	0.18 ± 0.24	0.23 ± 0.41	0.16 ± 0.30	0.16 ± 0.31	0.13 ± 0.25	0.17 ± 0.33	0.09 ± 0.22	0.11 ± 0.25	0.12 ± 0.23
	*F*	3.133	1.303	0.185	0.520	0.759	1.561	1.305	0.892	0.589
	*p*	0.046*	0.274	0.831	0.595	0.469	0.212	0.273	0.411	0.556
Marital status	Unmarried	0.08 ± 0.14	0.13 ± 0.26	0.11 ± 0.25	0.09 ± 0.26	0.08 ± 0.19	0.10 ± 0.21	0.04 ± 0.10	0.09 ± 0.20	0.08 ± 0.21
	Married	0.17 ± 0.27	0.22 ± 0.36	0.16 ± 0.30	0.16 ± 0.31	0.12 ± 0.24	0.16 ± 0.32	0.09 ± 0.20	0.13 ± 0.23	0.13 ± 0.23
	Divorced	0.21 ± 0.21	0.21 ± 0.17	0.18 ± 0.32	0.16 ± 0.21	0.12 ± 0.17	0.03 ± 0.07	0.01 ± 0.04	0.27 ± 0.58	0.18 ± 0.34
	*F*	2.874	1.353	0.796	1.052	0.557	1.535	2.619	2.190	1.225
	*p*	0.059	0.261	0.453	0.351	0.574	0.218	0.075	0.114	0.296
Only child	Yes	0.11 ± 0.17	0.16 ± 0.29	0.12 ± 0.26	0.12 ± 0.30	0.08 ± 0.16	0.11 ± 0.28	0.05 ± 0.12	0.11 ± 0.24	0.10 ± 0.21
	No	0.18 ± 0.29	0.22 ± 0.37	0.18 ± 0.31	0.15 ± 0.29	0.14 ± 0.27	0.17 ± 0.29	0.10 ± 0.21	0.14 ± 0.25	0.14 ± 0.25
	*F*	4.900	2.098	2.210	0.484	4.754	2.000	4.460	0.938	2.004
	*p*	0.028*	0.149	0.139	0.488	0.030*	0.159	0.036*	0.334	0.158
Education degree	Junior college	0.20 ± 0.29	0.30 ± 0.35	0.27 ± 0.35	0.22 ± 0.30	0.16 ± 0.26	0.22 ± 0.25	0.12 ± 0.21	0.19 ± 0.23	0.16 ± 0.26
	Undergraduate	0.14 ± 0.24	0.18 ± 0.34	0.14 ± 0.28	0.13 ± 0.29	0.11 ± 0.23	0.13 ± 0.29	0.07 ± 0.17	0.12 ± 0.25	0.12 ± 0.23
	Master degree or above	0.13 ± 0.14	0.13 ± 0.15	0.06 ± 0.11	0.07 ± 0.12	0.06 ± 0.09	0.06 ± 0.12	0.08 ± 0.18	0.12 ± 0.29	0.06 ± 0.17
	*F*	0.508	1.361	2.555	1.037	0.843	1.227	1.485	0.885	0.563
	*p*	0.602	0.256	0.080	0.356	0.432	0.295	0.229	0.414	0.570
Census register	Urban residence	0.12 ± 0.19	0.18 ± 0.29	0.12 ± 0.25	0.12 ± 0.29	0.08 ± 0.16	0.13 ± 0.28	0.06 ± 0.15	0.10 ± 0.20	0.10 ± 0.21
	Rural residence	0.18 ± 0.28	0.21 ± 0.37	0.17 ± 0.32	0.15 ± 0.29	0.14 ± 0.27	0.15 ± 0.29	0.09 ± 0.19	0.15 ± 0.28	0.14 ± 0.25
	*F*	3.014	0.522	1.550	0.750	4.795	0.395	1.989	2.492	1.432
	*p*	0.084	0.471	0.214	0.387	0.030*	0.531	0.160	0.116	0.233

^***^p < 0.001, **p < 0.01, *p < 0.05

### Influencing factors of pilot's HRQoL

The relationship between HRQoL, resilience, social support, and personality was examined using correlational analysis. Table 4 shows the analysis results. Resilience (strength, tenacity, and optimism), social support (family, friends, and other support), and personality (extraversion, agreeableness, conscientiousness, neuroticism, and openness to experience) were significantly correlated with HRQoL (physical function, physical role, bodily pain, and general health) (*p* < 0.05).

**Table 4:** Correlation analysis of physical health, resilience, social support and personality (*N* = 220)

		Physical function	Physical role	Bodily pain	General health
Personality	Extraversion	0.140*	0.349**	0.334**	0.473**
	Agreeableness	0.197**	0.266**	0.168*	0.453**
	Conscientiousness	0.286**	0.317**	0.219**	0.440**
	Neuroticism	-0.168*	-0.321**	-0.287**	-0.444**
	Openness	0.300**	0.337**	0.139*	0.341**
Resilience	Tenacity	0.267**	0.343**	0.299**	0.395**
	Strength	0.321**	0.391**	0.302**	0.442**
	Optimism	0.146*	0.219**	0.208**	0.196**
Social support	Family support	0.137*	0.168*	0.133*	0.193*
	Friend support	0.147*	0.186**	0.151*	0.196**
	Other support	0.150*	0.203**	0.147*	0.192**

^***^p < 0.001, **p < 0.01, *p < 0.05

The total SF-36 score was taken as the dependent variable and personality, resilience, and social support were taken as independent variables for the hierarchical regression analysis. The first layer was the three dimensions of social support, the second layer was the three dimensions of resilience, and the five dimensions of personality were included in the third layer. Table 5 shows the results. The regression equation is statistically significant and explains 33.6% of the total variation in physical health. The standardized regression coefficient of the strength (resilience) dimension to physical health was *β* = 0.519, *p* < 0.01. The standardized regression coefficient of the conscientiousness (personality) dimension to physical health was *β* = 0.186, *p* < 0.01.

**Table 5:** Hierarchical regression analysis of physical health (*N* = 220)

Layers	Factors	Non-standardized regression coefficient		*β*	*t*	*R*^*2*^	*ΔR*^*2*^	*F*
		*B*	*SE*					
First	Family support	-1.368	1.696	-0.195	-0.806	0.060	0.060	4.557^**^
	Friend support	0.859	1.774	0.119	0.484			
	Other support	2.252	1.924	0.310	1.171			
Second	Tenacity	0.005	0.735	0.001	0.007	0.250	0.191	11.861^**^
	Strength	4.672	1.251	0.519	3.736^**^			
	Optimism	-0.824	0.998	-0.062	-0.826			
Third	Extraversion	1.786	1.106	0.139	1.616	0.336	0.086	9.585^**^
	Agreeableness	-1.039	0.824	-0.109	-1.261			
	Conscientiousness	2.110	0.894	0.186	2.361^*^			
	Neuroticism	-1.207	0.818	-0.121	-1.476			
	Openness	0.468	0.836	0.044	0.560			

^***^p < 0.001, **p < 0.01, *p < 0.05

**Fig. 1:**
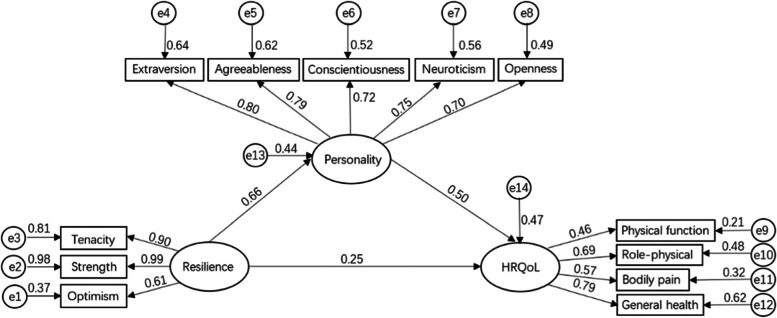
Pilots’ physical health influence factor model (*N* = 220)

To further explore the relationship between resilience, personality, and physical health of pilots, a structural equation model was constructed according to the above results. Figure 1 shows the model fitting degree parameters *χ*^*2*^*/ df* = 2.319, *p* < 0.01, NFI = 0.915, RFI = 0.890, IFI = 0.950, TLI = 0.935, CFI = 0.949, and RMSEA = 0.078. These values indicate that the model has a good fit. Resilience did not significantly predict HRQoL. The Sobel test result values were z = 3.56 > 1.96. Thus, personality fully mediates resilience and HRQoL. Resilience affects the HRQoL of pilots through personality factors.

The relationship between mental health, resilience, social support, and personality was described using correlation analysis. Table 6 shows the results. Resilience (strength, tenacity, and optimism), social support (family, friends, and other support), and personality (extraversion, agreeableness, conscientiousness, neuroticism, and openness to experience) were significantly correlated with mental health (somatization, obsessive symptoms, interpersonal sensitivity, depression, anxiety, hostility, terror, paranoia, and psychosis) (*p* < 0.05).

**Table 6:** The correlation analysis of mental health, resilience, social support and personality (*N* = 220)

		Somatization	Obsessive symptoms	Interpersonal sensitivity	Depression	Anxiety	Hostility	Terror	Paranoia	Psychosis
Personality	Extraversion	-0.345**	-.392**	-.403**	-0.405**	-0.420**	-0.370**	-0.297**	-0.284**	-0.395**
	Agreeableness	-0.312**	-0.347**	-0.366**	-0.379**	-0.359**	-0.351**	-0.373**	-0.346**	-0.404**
	Conscientiousness	-0.262**	-0.250**	-0.272**	-0.265**	-0.242**	-0.286**	-0.280**	-0.225**	-0.293**
	Neuroticism	0.394**	0.419**	0.480**	0.453**	0.458**	0.432**	0.367**	0.371**	0.446**
	Openness	-0.254**	-0.207**	-0.222**	-0.269**	-0.261**	-0.259**	-0.245**	-0.141*	-0.246**
Resilience	Tenacity	-0.227**	-0.311**	-0.327**	-0.393**	-0.311**	-0.264**	-0.224**	-0.247**	-0.269**
	Strength	-0.260**	-0.321**	-0.335**	-0.425**	-0.333**	-0.303**	-0.276**	-0.295**	-0.301**
	Optimism	-0.072	-0.163*	-0.208**	-0.265**	-0.215**	-0.096	-0.111	-0.172*	-0.142*
Social support	Family support	-0.196**	-0.137*	-0.204**	-0.139*	-0.211**	-0.150*	-0.205**	-0.172*	-0.244**
	Friend support	-0.192**	-0.150*	-0.172*	-0.180**	-0.205**	-0.180**	-0.212**	-0.163*	-0.229**
	Other support	-0.178**	-0.126	-0.175**	-0.139*	-0.206**	-0.146*	-0.200**	-0.159*	-0.230**

^***^p < 0.001, **p < 0.01, *p < 0.05

The total mental health score was taken as the dependent variable, and personality, resilience, and social support were taken as independent variables for the hierarchical regression analysis. The first layer was the three dimensions of resilience, the second layer was the three dimensions of social support, and the five dimensions of personality were included in the third layer. Table 7 displays the results. The regression equation is statistically significant and explains 29.7% of the total variation in mental health. The standardized regression coefficient of the friendship dimension of social support to mental health was *β* = -1.948, *p* < 0.05; The standardized regression coefficient of the neuroticism dimension of personality to mental health was *β* = 3.945, *p* < 0.01.

**Table 7:** Hierarchical regression analysis of mental health (*N* = 220)

Layers	Factors	Non-standardized regression coefficient		*β*	*t*	*R*^*2*^	*ΔR*^*2*^	*F*
		*B*	*SE*					
First	Tenacity	0.000	0.006	-0.017	-0.079	0.048	0.048	3.611
	Strength	-0.004	0.006	-0.148	-0.667			
	Optimism	0.004	0.007	0.132	0.548			
Second	Family support	-0.002	0.003	-0.107	-0.767	0.152	0.105	6.379
	Friend support	-0.010	0.005	-0.288	-1.948*			
	Other support	0.003	0.004	0.050	0.628			
Third	Extraversion	-0.007	0.005	-0.128	-1.440	0.297	0.144	7.970
	Agreeableness	-0.005	0.003	-0.128	-1.435			
	Conscientiousness	0.003	0.004	0.059	0.730			
	Neuroticism	0.013	0.003	0.332	3.945**			
	Openness	0.004	0.003	0.085	1.050			

^***^p < 0.001, **p < 0.01, *p < 0.05

**Fig. 2:**
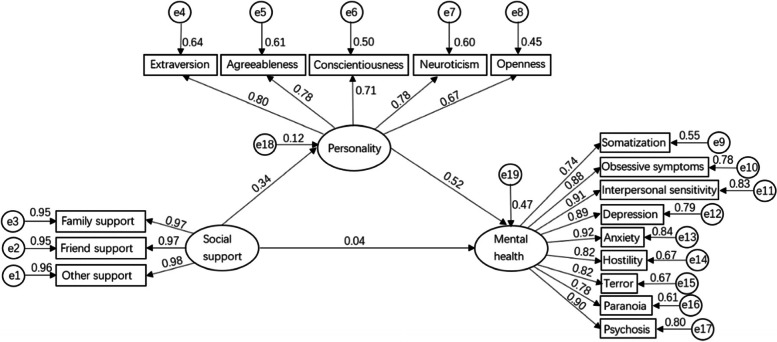
Pilots’ mental health influence factor model (*N* = 220)

To further explore the relationship between pilots’ social support, personality, and mental health, a structural equation model was constructed according to the above results. Figure 2 shows the model fitting degree parameters *χ*^*2*^*/ df* = 2.675,* p* < 0.01, NFI = 0.921, RFI = 0.907, IFI = 0.949, TLI = 0.940, CFI = 0.949, RMSEA = 0.087. These values indicate that the model has a good fit. Social support did not significantly predict mental health. Sobel test result values were *z* = 3.87 > 1.96. Therefore, personality shows a full mediation effect between resilience and mental health. Social support affects the mental health of pilots through personality factors.

## Discussion

In this study, we explored pilots’ health characteristics and other influencing factors, such as demographic data, personality traits, perceived social support, and resilience. We constructed a structural equation model of relevant factors.

As suggested in other research [13, 14], personality factors such as neuroticism and extraversion had a great impact on health at the psychological and behavioral levels. Neurotic individuals are more sensitive to negative emotions and also experience more adverse life events. They are also more likely to interpret events unfavorably, which has a deleterious impact on physical and psychological health [15]. In contrast, extroverted individuals tend to experience more positive life events. They also report more pleasant emotions on social occasions [16, 17]. It was suggested that actively integrating into social activities would help to release their emotions, which could be instrumental in relieving stress [18]. In addition, pilots' personality traits, such as emotional stability and adaptability, might significantly affect their mental health and flight performance [1]. Therefore, targeted intervention for pilots' personality characteristics could promote the improvement of their cognition, emotion, and behavior.

Social support is the understanding and utilization of assistance from important social partners such as family members, close friends, and others [19]. Many studies show that good social support will produce positive effects on health, while poor social support will lead to adverse outcomes [20–22]. Due to strict management, family separation, and a large number of tasks, social support is particularly essential for pilots’ health [23–25]. On the one hand, social support could improve the pilots’ ability to execute tasks. On the other hand, family support could provide pilots with emotional protection such as understanding and comfort. This support could reduce the impact of their negative emotional experiences and assist them in overcoming adversity [26, 27]. A recent study showed robust resilience, good social support, and a relaxed service environment predicts the post-retirement adaptability of pilots [28]. Studies have also shown that benign emotion regulation strategies and social relationships play a positive role in the retired life of pilots [19].

Significant life events have adverse effects on individual physiology and psychology, but some people still show adequate resilience [29]. Due to its potential impact on behavior, health, and HRQoL, resilience has gradually become a research hotspot [30, 31]. Resilience is an essential protective factor for the individual under stress, enhancing individual coping ability in a complex environment and supporting recovery from unpleasant emotional experiences [32, 33].

The physical function of pilots with a bachelor’s degree or above was significantly better than junior college pilots, perhaps due to different work positions. The majority of better-educated pilots reported that they could monitor their health more carefully and had more knowledge on how to protect themselves during sports and training and avoid excessive training, so as to maintain better physiological function. In terms of mental health, the somatization symptoms of pilots working five years or fewer were better than those of pilots employed for over five years. It is possible that along with increased service years, aggravated injuries and increased health sensitivity results in the growth of somatization symptoms. In addition, the somatization, anxiety, and terror levels of pilots who were only children were less severe than those of pilots raised with siblings, which might be related to the cultivation of child-rearing patterns and attachment types during childhood. Only children received more unconditional care from their parents. This care is conducive to the cultivation of safe attachment types. Children who shared parental care with siblings were more likely to develop contradictory attachment types, affecting mental health in adulthood.

The current study also found that urban pilots had better general health and anxiety than rural ones. Compared to ordinary jobs, pilots have to meet higher requirements for individual knowledge and cultural and practical skills. Urban pilots have more exposure to novel things starting in childhood. This experience might contribute to better adaptability and competence than rural pilots. These differences are reflected in levels of physical and mental health.

The results of our correlation analysis showed a significant correlation between pilots' HRQoL, personality, resilience, and social support. The results of hierarchical regression were more informative. Firstly, in the hierarchical regression of HRQoL, social support factors could not significantly predict HRQoL. After successively integrating resilience and personality traits, it was found that strength and conscientiousness played significant predictive roles in HRQoL. As suggested by other research, resilience helped individuals recover from anticipated threats, improving their work and life adaptability [34, 35]. Previous studies indicated that resilience could promote the recovery of individuals with coronary heart disease. It improved the adjustment and rehabilitation of children with chronic asthma [36, 37]. The value of strength shows that resilience directly impacts individual health. Study results also show that conscientiousness could significantly predict individual health. Our structural model showed that personality had a complete mediating effect between resilience and HRQoL. Therefore, in the health interventions with less resilient pilots, we should focus on less conscientious individuals, guide them to accept themselves, improve their personalities and adapt to life events.

On the other hand, the mental health hierarchical regression analysis shows that support from friends and neuroticism predict mental health levels. Support from friends is an important psychosocial factor affecting sleep quality. Due to the severe pressure of flying commercial planes and family separation, friendship is the primary social support for pilots. Strong support from friends provided pilots with an avenue for stress release and emotional disclosure, promoting mental health maintenance. In contrast with other personality traits, neuroticism reflects individual emotional stability. Pilots with high neuroticism scores were better able to manage their emotions according to various indicators of mental health. The structural equation model also showed that personality had a complete mediating effect on social support. Therefore, when intervening in pilots' mental health, we should focus on individuals who lack adequate support from friends and help them to improve their emotional management strategies and achieve emotional stability.

## Conclusions

We found a mediating effect of personality factors between resilience and the HRQoL of pilots. Personality factors also mediated the relationship between social support and pilots’ mental health. It is essential to address pilots’ workload and mental health, especially for those with less resilience and limited social support, to intervene in their mental health effectively.

## Data Availability

The datasets generated and analyzed during the current study are not publicly available due to confidentiality, but data is accessible from the corresponding author on reasonable request.

## References

[1] Dai J, Wang H, Yang L (2019). Emotional intelligence and emotional state effects on simulated flight performance. Aerosp Med Hum Perform.

[2] Ji SK, Yun YC (2020). Analysis of health problems among airline pilots in Korea (2016–2018). Korean J Aerosp Environ Med.

[3] Venus M  (2020). A survey of professional pilots health and wellbeing.

[4] Niederl T (2007). Investigations of cumulative psychic and psycho-physiological effects on flying staff examining short-haul operations. DLR Deutsches Zentrum fur Luft- und Raumfahrt e.V. Forschungsberichte.

[5] McAneney H, Tully MA, Hunter RF, Kouvonen A, Veal P, Stevenson M (2015). Individual factors and perceived community characteristics in relation to mental health and mental well-being. BMC Public Health.

[6] Blumenthal JA, Burg MM, Barefoot J, Williams RB, Haney T, Zimet G (1987). Social support, type A behavior, and coronary artery disease. Psychosom Med.

[7] Yaozhang, Dai, Xuewu, Li, Xin, Zhang, et al. Prevalence and predisposing factors for depressive status in Chinese patients with obstructive sleep Apnoea: a large-sample survey. PloS one. 2016;11(3):e0149939. 10.1371/journal.pone.0149939 PMC4774961 26934192

[8] Yu X, Zhang J, Yu XN, Zhang JX (2007). Factor analysis and psychometric evaluation of the Connor-Davidson resilience scale (CD-RICS) with Chinese people. Soc Behav Personal Int J.

[9] Xie Y, Li P, Xin Z, Min L. The psychometric evaluation of the Connor-Davidson resilience scale using a Chinese military sample. PloS one. 2016;11:e0148843. 10.1371/journal.pone.0148843 PMC4747584 26859484

[10] Carciofo R, Yang J, Song N, Feng D, Zhang K (2016). Psychometric evaluation of Chinese-Language 44-Item and 10-Item big five personality inventories, Including correlations with chronotype, mindfulness and mind wandering. PLoS ONE.

[11] Derogatis LR, Cleary PA (1977). Confirmation of the dimensional structure of the SCL-90: a study in construct validation. J Clin Psychol.

[12] Ware JE, Kosinski M, Dewey JE, Gandek B (2000). SF-36 health survey: manual and interpretation guide: Quality Metric Inc.

[13] Lamers SMA, Westerhof GJ, Kovács V, Bohlmeijer ET (2012). Differential relationships in the association of the Big Five personality traits with positive mental health and psychopathology. J Res Pers.

[14] Ozer DJ, Benet-Martínez V (2006). Personality and the prediction of consequential outcomes. Annu Rev Psychol.

[15] Suls J, Martin R (2005). The daily life of the Garden-Variety neurotic: reactivity, stressor exposure, mood spillover, and maladaptive coping. J Pers.

[16] Magnus K, Diener E, ujita FF, Pavot W (1993). Extraversion and neuroticism as predictors of objective life events: a longitudinal analysis. J Pers Soc Psychol.

[17] Pavot W, Diener E, Fujita F (1990). Extraversion and happiness. Personality Individ Differ.

[18] Watson David, Clark Lee, Mcintyre A, Hamaker CW (1992). Affect, personality, and social activity. J Pers Soc Psychol.

[19] Williston SK. Pathways to well-being in the lives of recently returning veterans: The roles of post-deployment social support and emotion regulation skills. Boston: University of Massachusetts; 2013.

[20] Lfb A, Tg B, Ib C, Tes D (2000). From social integration to health: Durkheim in the new millennium. Soc Sci Med.

[21] Sheldon, Cohen. Social relationships and health. Am psycho. 2004;59(8):676–84. 10.1037/0003-066X.59.8.676 15554821

[22] Skarupski KA, Parisi JM, Thorpe R, Tanner E, Gross D (2016). The association of adverse childhood experiences with mid-life depressive symptoms and quality of life among incarcerated males: exploring multiple mediation. Aging Ment Health..

[23] Grant-Vallone EJ, Donaldson SI (2001). Consequences of work–family conflict on employee well-being over time. Work Stress..

[24] Talya G, Joshua B, Christopher D, Neil G (2010). How communication with families can both help and hinder service members' mental health and occupational effectiveness on deployment. Mil Med..

[25] Kamphuis W, Venrooij W, Berg C (2012). A model of psychological resilience for the Netherlands armed forces.

[26] House JS, Kahn RL, Mcleod JD, Williams D. Measures and concepts of social support. New York: Social Support & Health Academic Press Inc.; 1985.

[27] Schaefer JA, Moos RH (1998). The context for posttraumatic growth: Life crises, individual and social resources, and coping.

[28] Cunningham Craig A, Weber Bryan A, Roberts Beverly L (2014). The role of resilience and social support in predicting postdeployment adjustment in otherwise healthy Navy personnel. Mil Med.

[29] Bartone PT (2006). Resilience under military operational stress: can leaders influence hardiness?. Mil Psychol.

[30] WHO/Europe. Mental health, resilience and inequalities. 2009.

[31] Casey George W (2011). Comprehensive soldier fitness: a vision for psychological resilience in the U.S. Army.. Army American Psychologist.

[32] Waugh CE, Fredrickson BL, Taylor SF (2008). Adapting to life’s slings and arrows: Individual differences in resilience when recovering from an anticipated threat. J Res Pers.

[33] Tugade MM, Fredrickson BL (2004). Resilient individuals use positive emotions to bounce back from negative emotional experiences. J Pers Soc Psychol.

[34] Waugh CE, Barbara L (2008). Adapting to life's slings and arrows: Individual differences in resilience when recovering from an anticipated threat.. J Res Pers.

[35] Leipold B, Greve W (2009). Resilience: a conceptual bridge between coping and development. Eur Psychol..

[36] Chan I, Lai J, Wong K (2006). Resilience is associated with better recovery in Chinese people diagnosed with coronary heart disease. Psychol Health.

[37] Kim DH, Yoo IY (2007). Factors associated with depression and resilience in asthmatic children. J Asthma.

